# Supplementary material for: Cluster-type analogue memristor by engineering redox dynamics for high-performance neuromorphic computing
Source: Nat Commun. 2022 Jul 12;13:4040. doi: 10.1038/s41467-022-31804-4 (PMC9279478; doi:10.1038/s41467-022-31804-4)
Supplement: Supplementary file 1 — Supplementary Information [file 41467_2022_31804_MOESM1_ESM.pdf]

Supplementary Information

Cluster-type analogue memristor by engineering redox dynamics for high-performance neuromorphic computing

Jaehyun Kang[1,2], Taeyoon Kim[1], Suman Hu[1], Jaewook Kim[1], Joon Young Kwak[1], Jongkil Park[1], Jong Keuk Park[1], Inho Kim[1], Suyoun Lee[1], Sangbum Kim[2], and YeonJoo Jeong[1] *

[1]*Center for Neuromorphic Engineering, Korea Institute of Science and Technology, Seoul, Republic of Korea*

[2]*Department of Materials Science and Engineering, Seoul National University, Seoul, Republic of Korea*

* Correspondence to jeongyeonjoo@kist.re.kr

Supplementary Figures

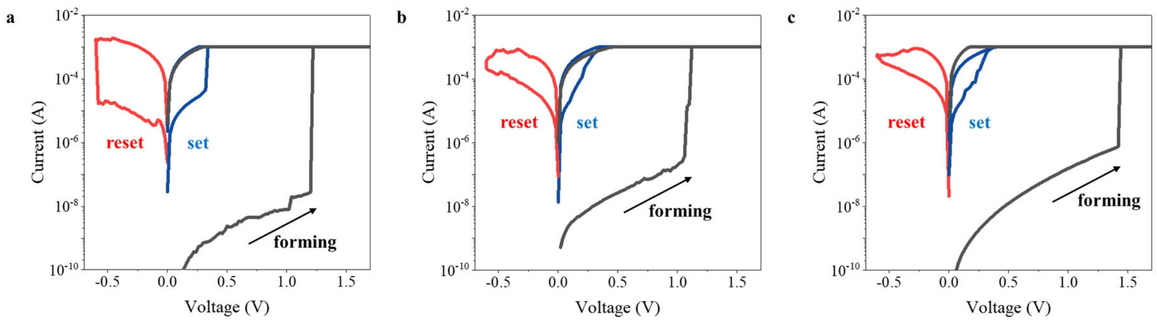

Supplementary Fig. 1 Quasi-static current-voltage (*I*-*V*) switching characteristics of the forming process with the following set and reset processes. The electrical forming process with a compliance current of 1 mA is required for **a** a-Si (pristine), **b** a-Si (densified), **c** and $Ti_{4.8\%}$:a-Si devices. The observed forming voltages are found around 1.2~1.5 V, which is highly desirable for memristor crossbar array integration, preventing the permanent breakdown of the memristor devices due to its low forming voltage (<2 V). We speculate that the low forming voltage originates from the thin film thickness (~7 nm) of the switching layer of the device.

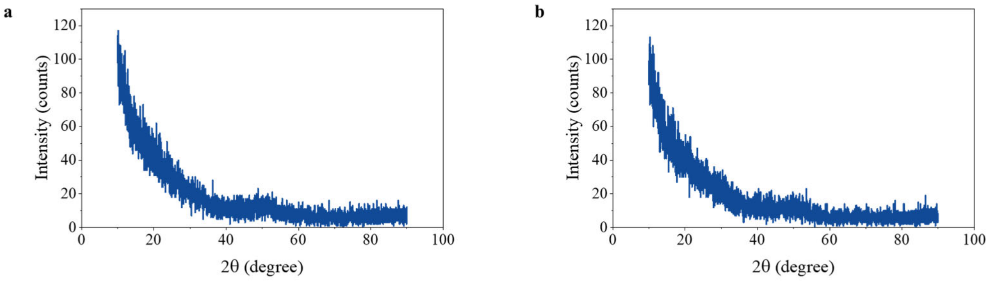

Supplementary Fig. 2 X-ray diffraction (XRD) 2θ plots of a-Si (densified) and $Ti_{4.8\%}$:a-Si layers. Both films, **a** a-Si (densified) and **b** $Ti_{4.8\%}$:a-Si, are deposited at 350 °C substrate temperature with a post rapid thermal annealing (RTA) process at 350 °C for 5 minutes, and none of the crystalline phases is detected.

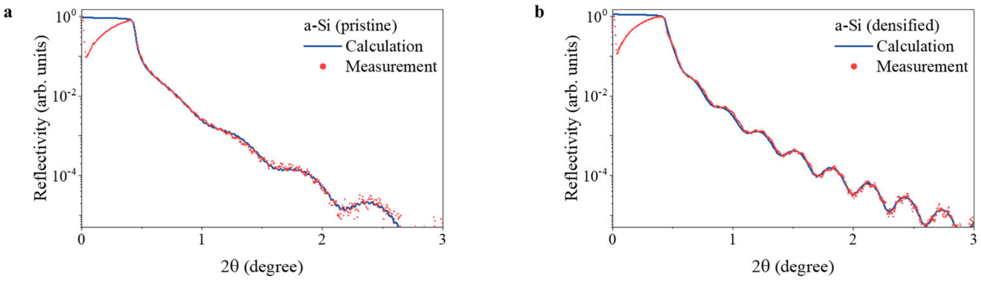

Supplementary Fig. 3 X-ray reflectometry (XRR) spectra of a-Si (pristine) and a-Si (densified) layers. a The pristine a-Si film was deposited at 20 W and room substrate temperature. **b** The densified a-Si film was deposited at 70 W at 350 °C substrate temperature with a post RTA process at 350 °C for 5 minutes. The pristine a-Si film showed 1.95 g cm^{-3}, and the densified a-Si film showed 2.26 g cm^{-3} amorphous phase atomic density.

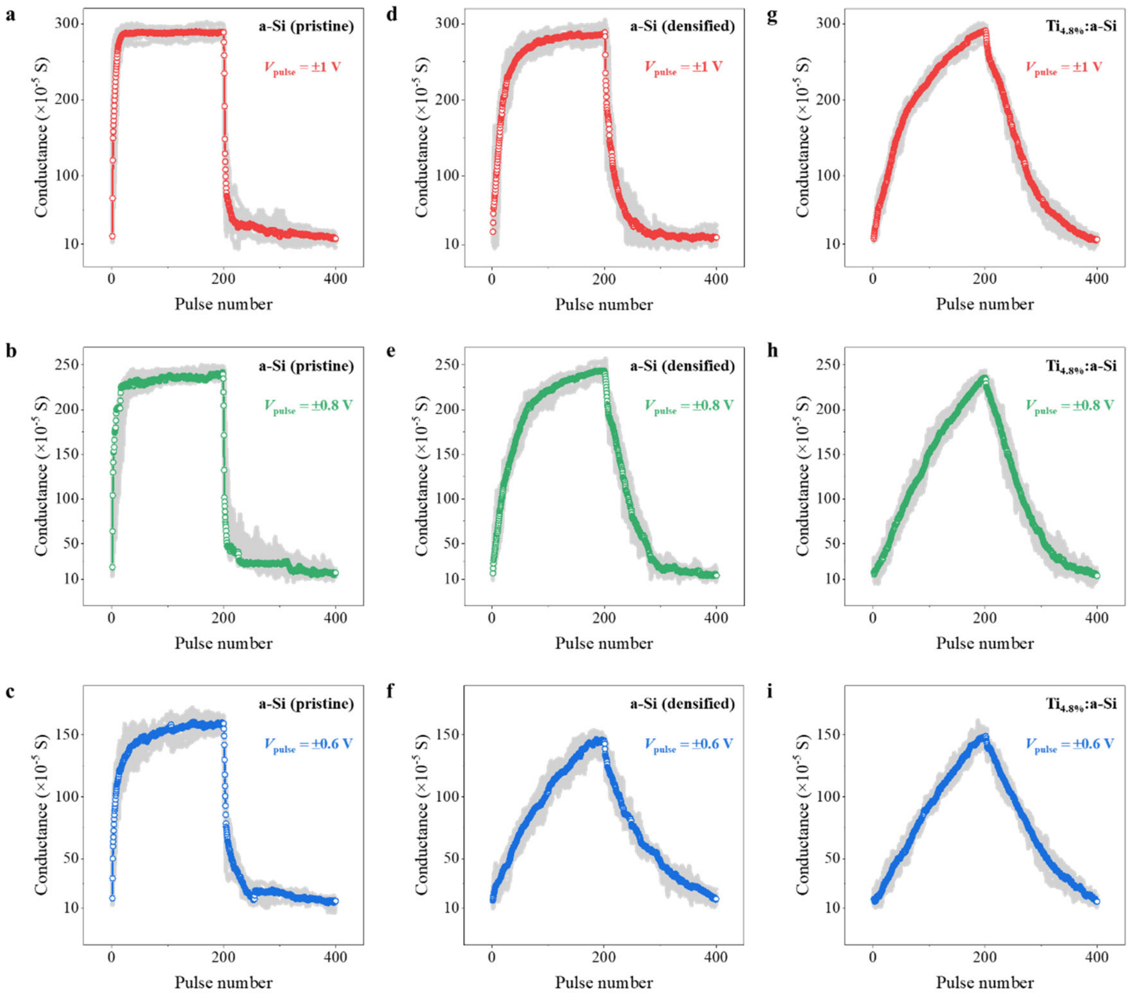

Supplementary Fig. 4 Ten-cycle analogue conductance updates measured under three different pulse amplitudes. a-c a-Si (pristine) device, **d-f** a-Si (densified) device, and **g-i** $Ti_{4.8\%}$:a-Si device. Blue, green, and red plots represent the average of ten-P/D-cycle (grey) for each device, measured under three different pulse conditions of 0.6/-0.6 V, 0.8/-0.8 V, and 1/-1 V with 1 μs duration, respectively. The conductance was measured with a read pulse (0.1 V, 1 μs) after each programming pulse.

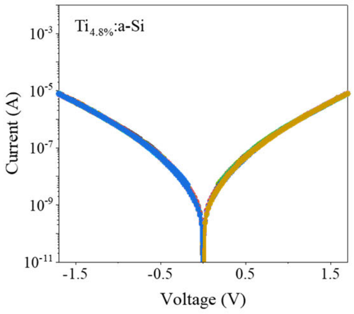

Supplementary Fig. 5 Multiple quasi-static current-voltage (*I*-*V*) sweeps of the $Ti_{4.8\%}$:a-Si memristor without an Ag layer in the top electrode. To verify Ag-based resistive switching in the $Ti_{4.8\%}$:a-Si device, we deposited a 50 nm single Pt layer in the top electrode rather than 10 nm Ag and 40 nm Pt multilayer. We repetitively swept from 0 → +1.7 V and 0 → -1.7 V for 30 cycles, considering the forming voltage of Ag-based $Ti_{4.8\%}$:a-Si memristor (forming voltage of ~1.5 V). Resistive switching was not observed during the DC cycle measurement, showing that the silicide bonding of Ti only assures Ag-based analogue switching.

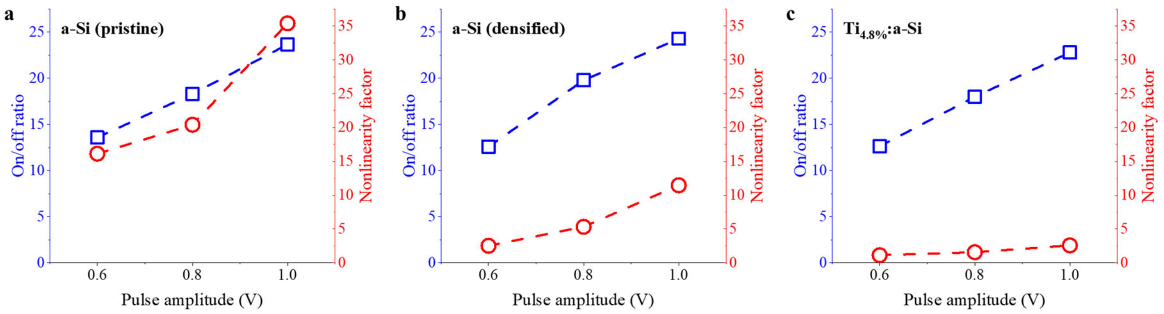

Supplementary Fig. 6 Average nonlinearity factor and on/off ratio of ten-P/D-cycle under three different pulse amplitudes. a a-Si (pristine) device, **b** a-Si (densified) device, and **c** $Ti_{4.8\%}$:a-Si device. The trade-off relationship between the nonlinearity factor and on/off ratio is clearly demonstrated in a-Si (pristine) and a-Si (densified) devices. However, the cluster-type $Ti_{4.8\%}$:a-Si memristor maintained a low nonlinearity factor in large pulse amplitude with a high on/off ratio.

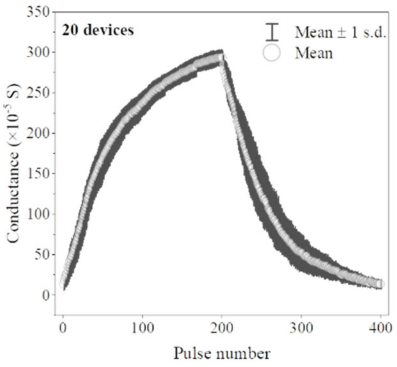

Supplementary Fig. 7 Device-to-device variation of the $Ti_{4.8\%}$:a-Si memristor performed under the 1.1/-1.1 V, 1 μs pulse condition. Analogue conductance update curves of 20 devices are plotted by mean and mean ±standard deviation (s.d.). Since the conductance level is not yet saturated, the size of the conductance update is the largest. Accordingly, the s.d. values are larger in the earlier pulse number in potentiation and depression.

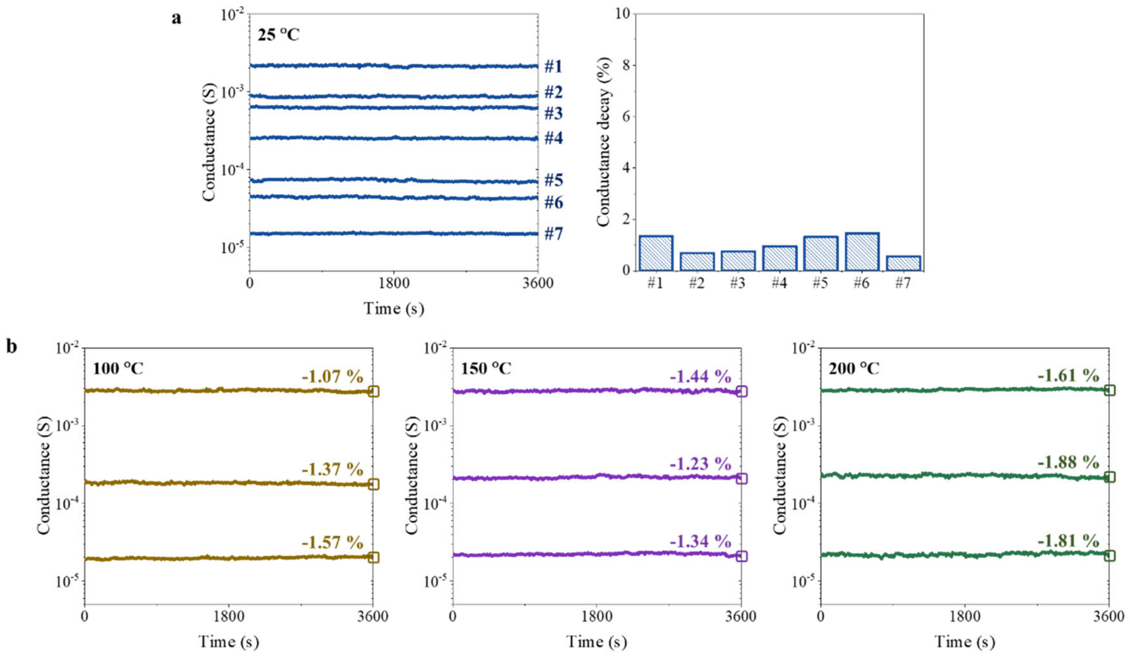

Supplementary Fig. 8 Analogue data retention test with raised the temperature of $Ti_{4.8\%}$:a-Si device for 1 h. a The device is tested at various multi-conductance levels at room temperature, and the conductance decay (%) at each conductance level. **b** The retention test of low-, mid-, and high-conductance levels with the raised temperature at 100 °C, 150 °C, and 200 °C and the corresponding conductance decay (%) values. The devices were programmed to a specific conductance state before the retention test, measured by 0.1 V read bias.

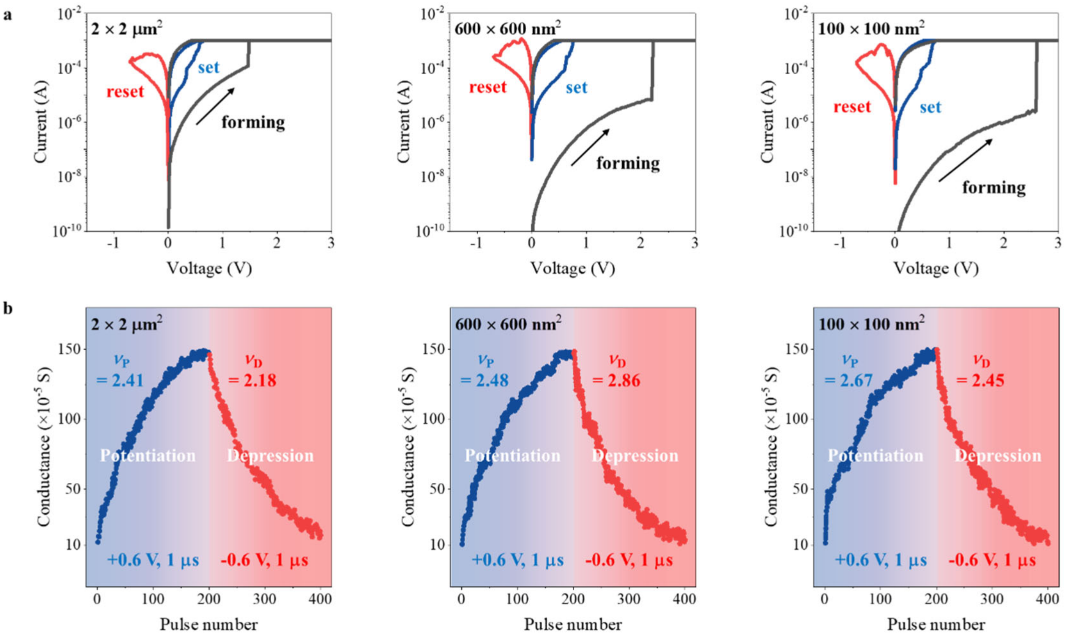

Supplementary Fig. 9 The scaling effect of junction size in $Ti_{4.8\%}$:a-Si device switching behaviour (2 × 2 μm^2 ~ 100 × 100 nm^2). a Quasi-static current-voltage (*I*-*V*) switching characteristics of the forming process with the following set and reset processes for the 2 × 2 μm^2 ~ 100 × 100 nm^2 junction devices. **b** Analogue conductance updates of the 2 × 2 μm^2 ~ 100 × 100 nm^2 junction devices under 0.6/-0.6 V pulse condition with 1 μs duration for potentiation and depression.

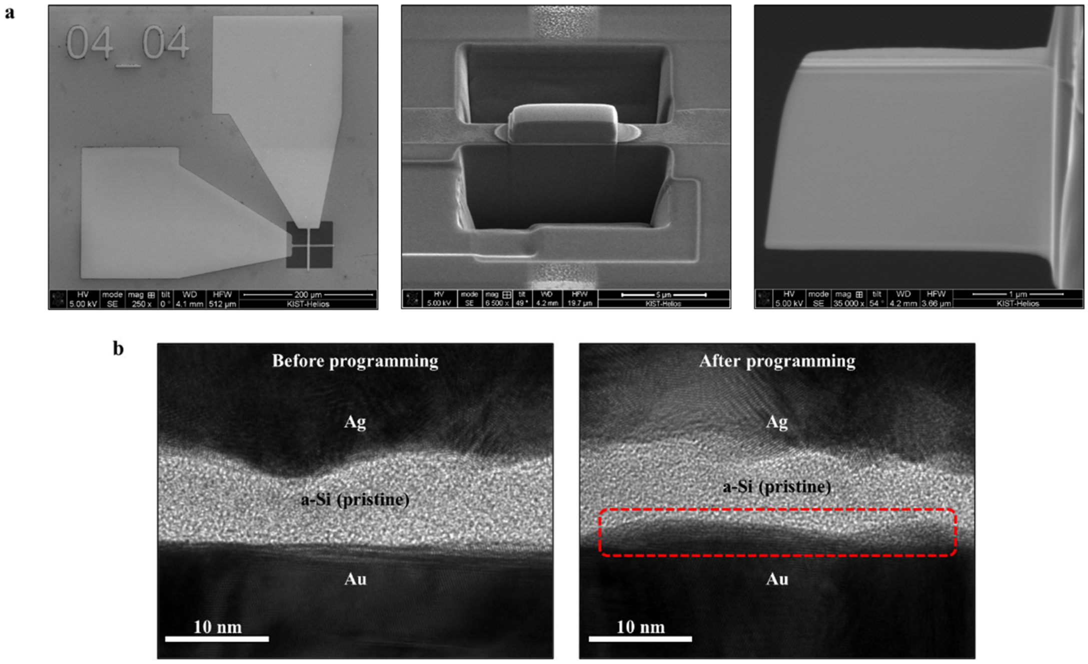

Supplementary Fig. 10 Electron microscopy specimen preparation and microstructure analysis of a-Si (pristine) memristor. a Scanning electron microscopy (SEM) images of electron microscopy specimen preparation using the focused ion beam (FIB) system. **b** Cross-sectional transmission electron microscopy (TEM) images of a-Si (pristine) memristor taken before/after programming. The Ag filament started to grow at the surface of the inert electrode (Au), which is the conventional filament growth dynamics of conductive-bridge random access memory (CBRAM), resulting in abrupt resistive switching.

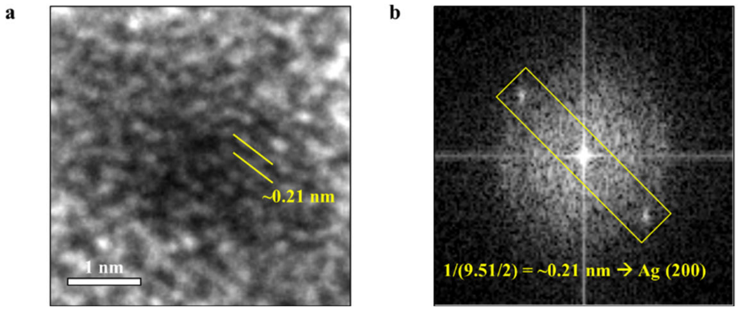

Supplementary Fig. 11 High-resolution transmission electron microscopy (HRTEM) image of the nucleated Ag-cluster inside $Ti_{4.8\%}$:a-Si switching layer. a HRTEM image of the nucleated Ag-cluster. **b** Corresponding fast Fourier transform results. The lattice fringes in the image are successfully resolved to Ag (200) crystal planes. The presence of Ag-cluster was clearly identified in the switching layer region for its Ti-assisted reduction mechanism.

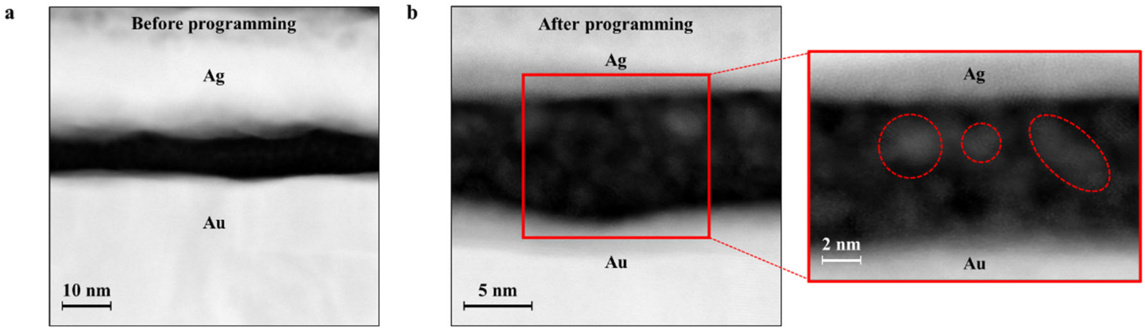

Supplementary Fig. 12 Scanning transmission electron microscopy (STEM) images of $Ti_{4.8\%}$:a-Si memristor taken before/after programming. a Before programming. **b** After programming (right inset: the high-resolution image of an after programming the device). Red circles indicate the Ag-clusters nucleated inside the $Ti_{4.8\%}$:a-Si switching layer.

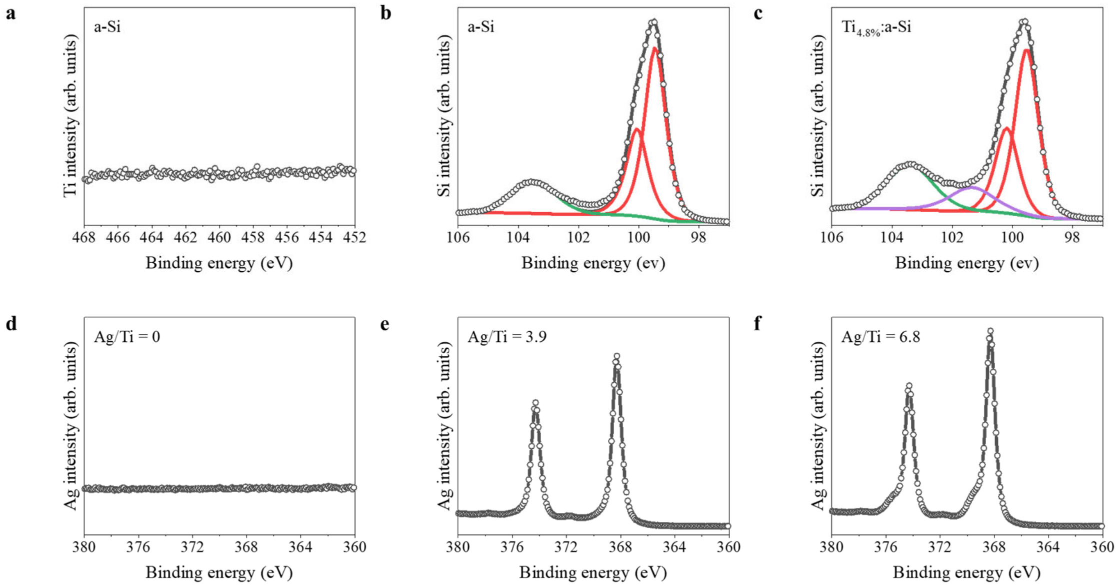

Supplementary Fig. 13 X-ray photoelectron spectroscopy (XPS) results of a-Si, $Ti_{4.8\%}$:a-Si, and Ag-Ti-Si co-deposited films. a Ti and **b** Si for the a-Si film**. c** Si for the $Ti_{4.8\%}$:a-Si film. **d-f** Ag for Ag-Ti-Si co-deposited films with Ag/Ti ratios of 0, 3.9, and 6.8. All films are deposited in the same condition as the device fabrication procedure.

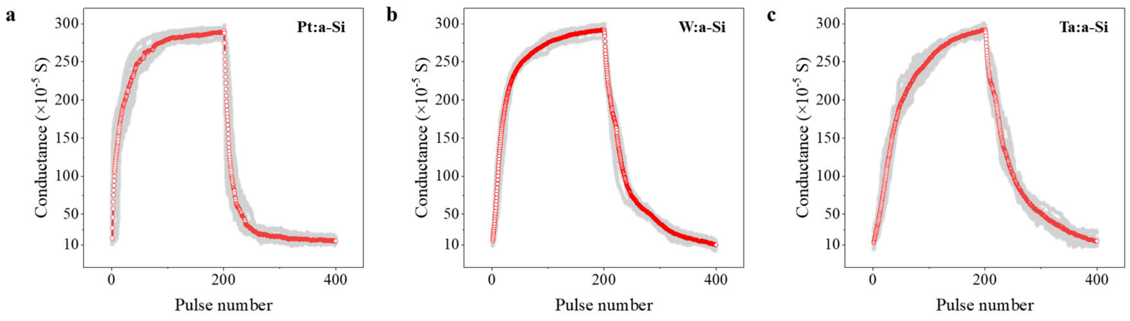

Supplementary Fig. 14 Ten-cycle analogue conductance updates measured under the 1/-1 V, 1 μs pulse condition. a Pt:a-Si device, **b** W:a-Si device, and **c** Ta:a-Si device. The red plot represents the average of ten-P/D-cycle (grey) for each device. The conductance was measured with a read pulse (0.1 V, 1 μs) after each programming pulse.

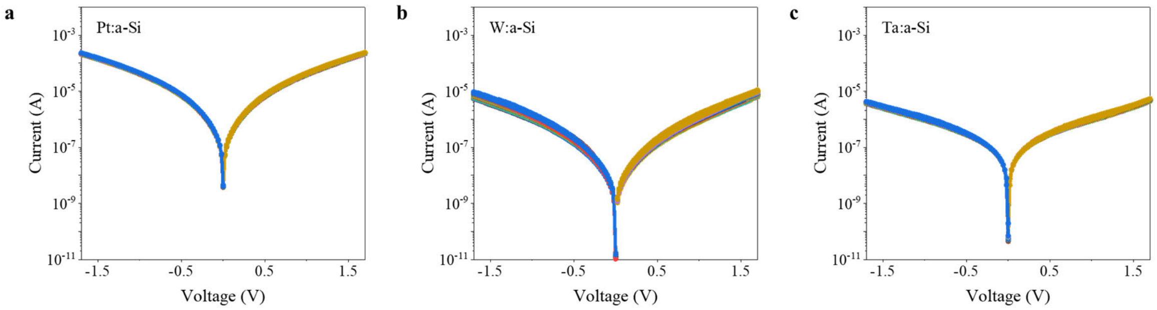

Supplementary Fig. 15 Multiple quasi-static current-voltage (*I*-*V*) sweeps of the M:a-Si (M = Pt, W, and Ta) memristors without an Ag layer in the top electrode. a Pt:a-Si device, **b** W:a-Si device, and **c** Ta:a-Si device. To verify Ag-based resistive switching in M:a-Si (M = Pt, W, and Ta) devices, we deposited a 50 nm single Pt layer in the top electrode rather than 10 nm Ag and 40 nm Pt multilayer. We repetitively swept from 0 → +1.7 V and 0 → -1.7 V for 30 cycles, considering the forming voltage of Ag-based M:a-Si (M = Pt, W, and Ta) memristors (forming voltage of ~1.5 V). Resistive switching was not observed during the DC cycle measurement that silicide bonding of Pt, W, and Ta assure only Ag-based analogue switching.

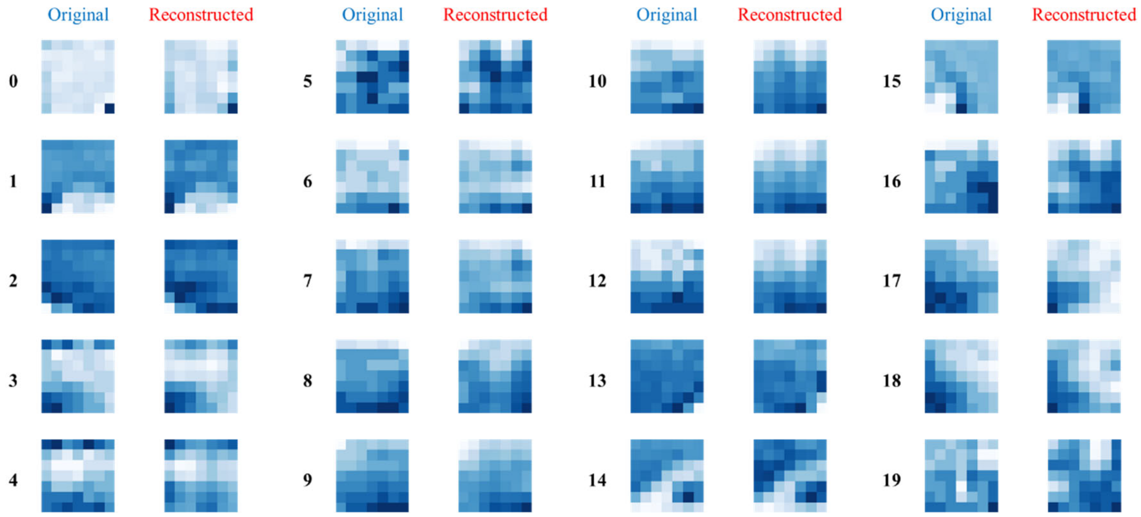

Supplementary Fig. 16 Examples of blue colour filtered original input images and the reconstructed images after the network training. The threshold, $\lambda = 0.1$, is used.

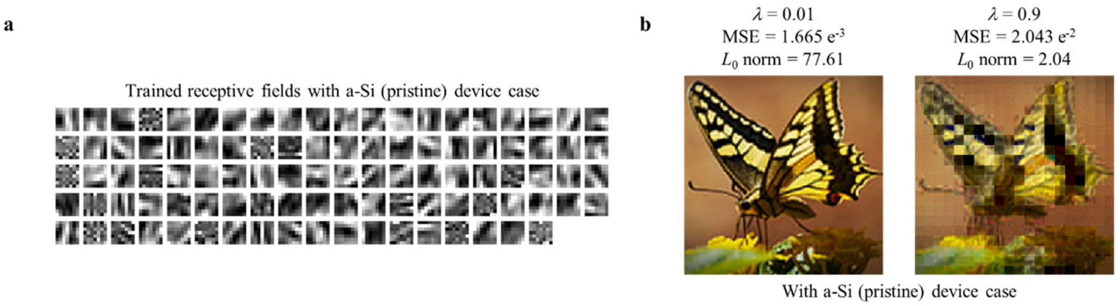

Supplementary Fig. 17 Feature extraction task of sparse coding simulation using the measured a-Si (pristine) device characteristics. a Trained receptive fields through the stochastic gradient descent and locally competitive algorithm (LCA) algorithm after 50 iterations. The identical natural images in Fig. 5a (main text) are used for training receptive fields. **b** Reconstructed images after sparse coding simulation with threshold, $\lambda = 0.01/0.9$, and resultant L_0 norm.

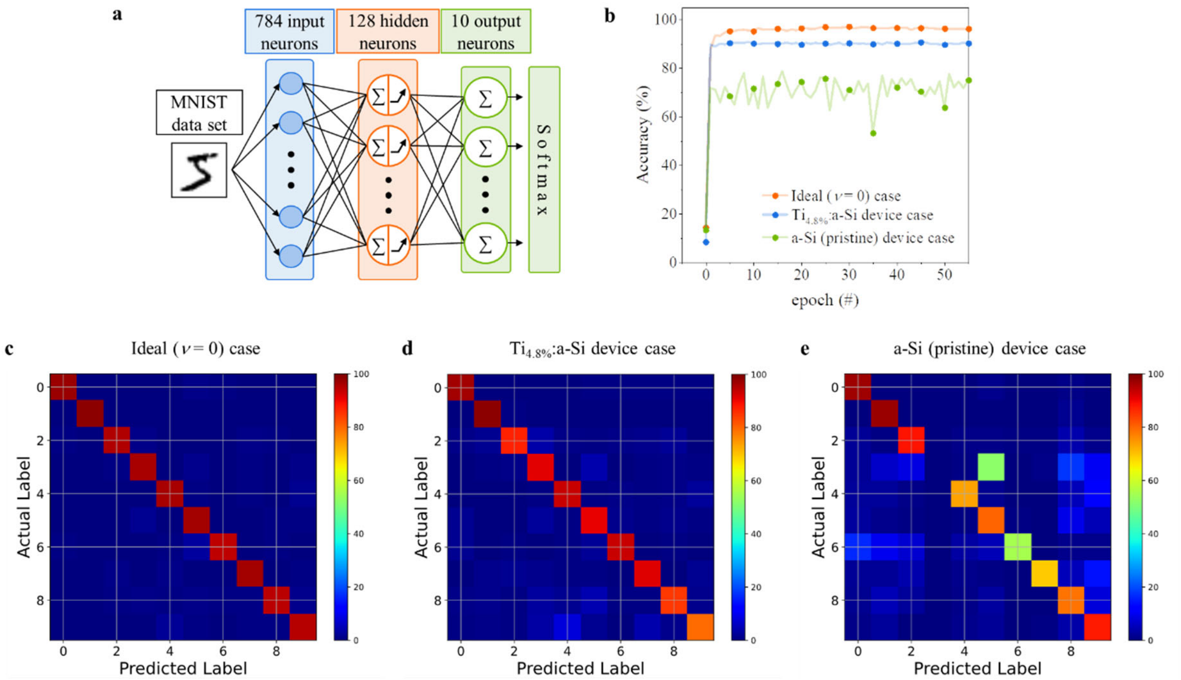

Supplementary Fig. 18 Modified National Institute of Standards and Technology (MNIST) recognition simulation of the memristor-based neural network. a Schematic illustration of the multilayer perceptron (MLP) neural network with 784 input layer neurons, 128 hidden neurons, and 10 output neurons. **b** Accuracy evolution of training epochs with the ideal (ν = 0) case, $Ti_{4.8\%}$:a-Si, and a-Si (pristine) device cases. Confusion-matrix classification diagrams of the MNIST dataset for **c** ideal (ν = 0) case, **d** $Ti_{4.8\%}$:a-Si, and **e** a-Si (pristine) device cases. With our experimental memristor device characteristics, we performed a deep neural network simulation for supervised learning of MNIST classification task. We utilized the MLP algorithm with the stochastic gradient descent method during the whole training epochs, and the ReLU activation function was adopted to perform nonlinear encoding. In the output layer, the Softmax function generates the normalized probability distribution of each output value. Supplementary Fig. 20b presents the results of the training accuracy of the MNIST classification task under the ideal (ν = 0) case, $Ti_{4.8\%}$:a-Si, and a-Si (pristine) device cases. Supplementary Fig. 20c-e displays confusion-matrix diagrams for the MNIST dataset in

which each pixel is closer to red, the higher the predicted accuracy of a single number digit. Our simulation result proves that the neural network based on the $Ti_{4.8\%}$:a-Si device case can function close to the ideal ($\nu = 0$) case, achieving high classification accuracy.
